# Concomitant Ulnar Styloid Fractures in Distal Radius Osteosynthesis Does Not Impact Radiographic Outcomes, Ulnar Sided Symptoms and Patient Outcomes

**DOI:** 10.5704/MOJ.2303.017

**Published:** 2023-03

**Authors:** KC Wong, MWF Wu, QJJ Zai, MK Wong, TS Howe, SBJ Koh, H Soeharno

**Affiliations:** 1 Department of Orthopaedic Surgery, Singapore General Hospital, Singapore; 2 Department of Urology, Gleneagles Hospital, Singapore; 3 Intensive Care Unit, Mount Elizabeth Novena Hospital, Singapore; 4 Department of Orthopaedic Surgery, Sengkang General Hospital, Singapore

**Keywords:** wrist, distal radius fracture, ulnar styloid, outcome, triangular fibrocartilage complex

## Abstract

**Introduction:**

Current literature reports varied significance of ulnar styloid fractures (USF) associated with distal radius fractures. Our study assesses the role of ulnar styloid fractures and fragment size in surgically managed distal radius fractures.

**Materials and methods:**

We reviewed patients who underwent surgical fixation of distal radius fractures between January 2004 to June 2006. Patients were divided into those with (Group 1) and without (Group 0) USFs. Post-operative radiographic parameters, clinical outcomes and overall wrist function were analysed. Outcomes included ulnar-sided wrist pain, extensor carpi ulnaris (ECU) tendinitis, triangular fibrocartilage complex (TFCC) grind test, distal radioulnar joint (DRUJ) instability and pain. Overall wrist function was assessed with range of motion and Disabilities of the Arm, Shoulder and Hand (DASH) score.

**Results:**

Our study cohort included 31 males and 23 females, and 38.9% of these patients had concomitant USFs. There was no difference in terms of demographic data and fracture configuration between groups. Radiographic parameters were similar, except for palmar tilt, which was significantly higher in Group 1 (4.6º vs 9.4º, p=0.047). At 24 months, there were no differences in clinical outcomes and overall wrist function. A sub-group analysis showed that mean USF fragment size was larger in patients with a positive TFCC grind test (3.9mm vs 7.3mm, p=0.033).

**Conclusion:**

The presence of USFs in surgically managed distal radius fractures does not compromise clinical and functional outcome. Similarly, the size of USFs does not impact clinical and functional outcome but is associated with the presence of a positive TFCC grind test.

## Introduction

Age, amongst selected radiological parameters, has been well established as an important indicator for surgery when managing patients with a distal radius fracture, as recent studies have shown no improvement in outcomes after distal radius fixation in geriatric patients^1,2^. However, the consensus on concomitant ulnar styloid fixation remains unclear.

There is a high prevalence of an ulnar styloid fracture seen in distal radius fractures, with studies reporting occurrences as common as more than 50% of cases^3^. However, despite reporting that ulnar styloid fractures result in poorer outcomes of distal radius fractures, studies did not show that fixation of ulnar styloid fractures yielded superior outcomes^4^. Hence, a large proportion of associated ulnar styloid fractures have been conservatively managed as some studies showed that they had comparable outcome scores, range of motion, grip strength and ulnar-sided symptoms when compared to distal radius fractures without ulnar styloid involvement^3,5-7^.

Current literature seems to report varied significance of ulnar styloid fractures associated with distal radius fractures, and there is a paucity of studies investigating the effects of ulnar styloid fracture fragment size in patients with surgically managed distal radius fractures. Our study aims to assess the importance of ulnar styloid fractures and ulnar styloid fragment size in surgically managed distal radius fractures. We hypothesise that the fragment size of an ulnar styloid fracture, rather than its presence, has implications in the outcomes of patients with surgically managed distal radius fractures.

## Materials and Methods

This was a prospective review of a retrospective cohort of patients who underwent surgical fixation of distal radius fractures in our institution between January 2004 to June 2006. Inclusion criteria were patients aged 18 years or above requiring open reduction internal fixation of a distal radius fracture. Open fractures, poly-trauma with other concomitant upper extremity injuries, fractures involving the ulnar shaft, or surgically managed ulnar styloid fractures were excluded from this study. Institutional review board approval was obtained with the need for informed consent waived. We reviewed the clinical records of 54 patients and collected demographic data including age and gender. The AO classification of each fracture, based on the distal radius classification, was also documented^8^. These patients were then divided into those with (Group 1) and without (Group 0) ulnar styloid fractures. Treatment of distal radius fractures in our institution were left to the discretion of individual surgeons, and all surgeries in this study were performed by fully trained orthopaedic surgeons.

**Fig. 1:**
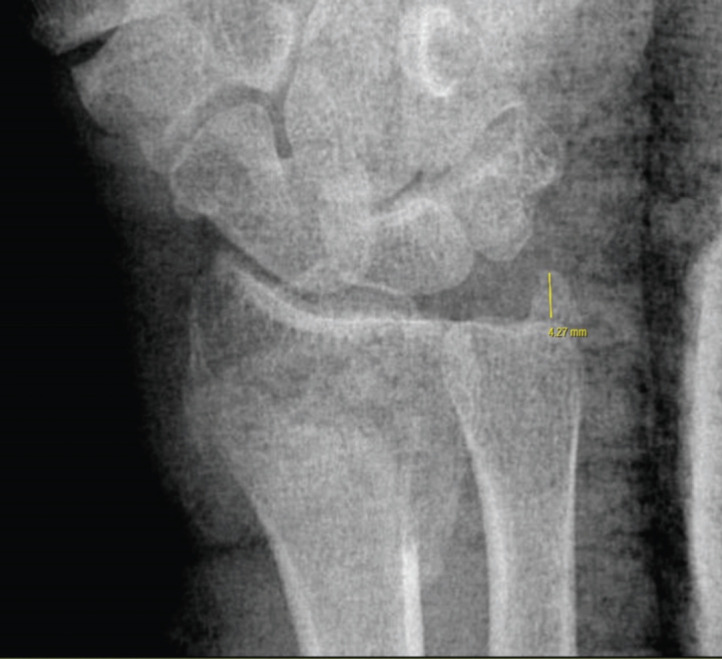
Measurement of an ulnar styloid fracture fragment on pre-operative radiograph.

**Fig. 2:**
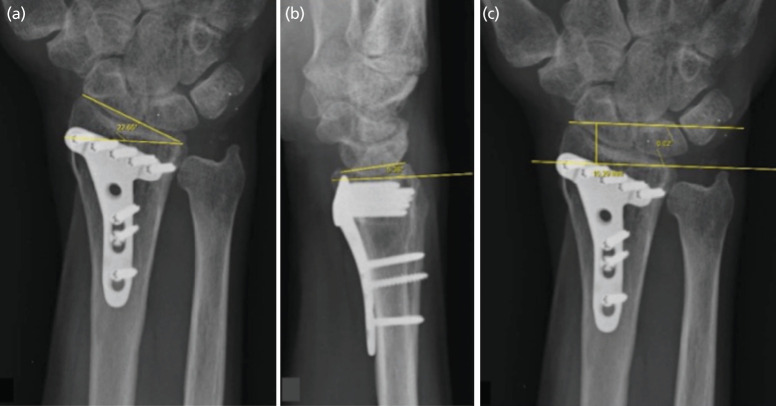
(a) Degree of radial inclination post-fixation. (b) Degree of palmar tilt post-fixation. (c) Amount of radial shortening post-fixation

Standard anteroposterior and lateral views on plain radiographs of the injured wrist before surgery, post-surgery, and at six months after surgical intervention were reviewed. The size of the ulnar styloid fragment was measured using the pre-operative radiograph, measuring the distance from the tip of the fragment to the base of the fragment (Fig. 1). We measured the radial inclination, dorsal comminution, ulnar variance, palmar tilt, radial shortening, and intra-articular step-off on radiographs taken six months post-operatively (Fig. 2). All radiographic parameters were measured by two independent assessors.

We compared outcomes between both groups at 24 months post-surgery, including ulnar-sided wrist pain, extensor carpi ulnaris (ECU) tendinitis, and the TFCC grind test for clinical evidence of possible TFCC injury. Presence of ulnar-sided wrist pain was recorded when foveal pain was elicited on palpation. Pain over DRUJ was elicited on palpation of involved DRUJ during clinical examination. DRUJ instability was assessed in terms of the “piano key” sign. ECU tendinitis was assessed clinically by localised swelling with tenderness from the ulnar styloid to the base of the 5th metacarpal, in the presence of pain reproduced on resisted active extension and ulnar deviation of the wrist. Any weakness denoting possible rupture was also noted. A positive TFCC grind test is achieved when wrist in maximum ulnar deviation, and axial stress produces ulnar wrist pain during passive supination-pronation^9^. Overall wrist function was assessed by an independent assessor in terms of range of motion and the Disabilities of the Arm, Shoulder and Hand (DASH) score. DASH questionnaires evaluate patient-reported global disabilities of the upper extremity via a 30-item questionnaire, where patients rate difficulties in activities of daily living on a 5-point scale. The test score ranges from 0 to 100, with a higher score indicating a higher level of disability. The DASH questionnaire is not specific to the wrist joint but is the most commonly used outcome measure to assess the wrist joint^10-13^.

All patients were independently assessed in an outpatient clinic by two trained Orthopaedic residents. Pearson chi-squared test was used to compare categorical data, while the student’s t-test was used to analyse continuous variables. Statistical analyses were performed with SPSS v23.0 [SPSS Inc, Chicago, IL, USA] software and statistical significance was defined as p<0.05.

## Results

Our study cohort included 31 males and 23 females with a mean age of 50.9 years (range, 18-88; SD, 16.5). 21 out of the 54 (38.9%) patients had concomitant ulnar styloid fractures. The follow-up rate was 100% at 2 years. When comparing both groups, there was no significant difference in terms of age (52.6 years vs 48.1 years, p=0.327), gender distribution (19:14 vs 12:9, p=0.598) and fracture configuration based on the AO classification (p=0.389) (Table I).

**Table I:** Cohort demographic data.

		Group 0 (n=33)	Group 1 (n=21)	p– value
Median Age (years)		52.6	48.1	0.327
Gender (M:F)		19:14	12:9	0.598
AO (distal radius) Classification	A	13	5	0.389
	B	10	7	
	C	10	9	

On the last available radiographs at six months post-surgery, all distal radius fractures had united. There was no significant difference in radial inclination (21.2º vs 23.0º, p=0.571), positive ulnar variance (39.4% vs 28.6%, p=0.561), and the presence of dorsal comminution (51.5% vs 71.4%, p=0.274) between the two groups. However, palmar tilt was significantly higher in Group 1 (4.6º vs 9.4º, p=0.047). Both groups had minimal radial shortening and intra-articular step-off (Table II). Most ulnar styloid fractures did not achieve bony union on follow-up radiographs.

**Table II:** Radiological assessments between Groups 0 and 1.

	Group 0 (n=33)	Group 1 (n=21)	p– value
Radial inclination	21.2°	23.0°	0.571
Dorsal comminution	51.5%	71.4%	0.274
Ulnar variance (positive)	39.4%	28.6%	0.561
Palmar tilt	4.6°	9.4°	0.047
Radial shortening	Minimal	Minimal	-
Intra-articular step off	Minimal	Minimal	-

At 24 months, the presence of ulnar styloid fractures had no impact on ulnar-sided wrist pain (p=0.331), TFCC grind test (p=0.917), distal radioulnar joint instability (p=0.957), and pain over the distal radioulnar joint (p=0.225). None of the patients had clinical evidence of ECU rupture, but there was a tendency towards ECU tendinitis in Group 1 (6.1% vs 23.8%, p=0.058), but this did not achieve statistical significance. There was no significant difference in range of motion and DASH scores (8.0 vs 5.9, p=0.474) (Table III).

**Table III:** Clinical outcomes between Groups 0 and 1..

Clinical Outcomes	Group 0 (n=33)	Group 1 (n=21)	p– value
Mean DASH scores (SD)	8.0 (12.9)	5.9 (7.1)	0.474
Ulnar-sided wrist pain	3.03%	4.76%	0.331
TFCC grind test	15.2%	19.0%	0.917
DRUJ instability	9.09%	9.52%	0.957
Pain over DRUJ	12.1%	23.8%	0.225
ECU tendinitis	6.1%	23.8%	0.058
Mean range of motion (SD)			
Flexion	43.8 (20.0)	43.0 (16.2)	0.873
Extension	50.5 (20.5)	52.0 (14.0)	0.774
Ulnar deviation	32.2 (13.4)	36.9 (13.2)	0.221
Radial deviation	16.5 (7.2)	19.0 (10.6)	0.304
Supination	85.9 (8.6)	81.0 (19.1)	0.275
Pronation	85.5 (8.7)	83.1 (16.7)	0.498

A sub-group analysis was also performed on subjects in Group 1, with the list of USF sizes of each patient listed in Table IV. We found that the size of the ulnar styloid fracture fragment had no impact on ulnar sided wrist pain (p=0.518), pain in the distal radioulnar joint (p=0.829), ECU tendinitis (p=0.603), and DRUJ instability (p=0.952). However, the mean ulnar styloid fragment size was significantly larger in patients with a positive TFCC grind test (3.9mm vs 7.3mm, p=0.033) (Table V). Size of ulnar styloid fragment also had no impact on DASH score (9.4 vs 6.6, p=0.648) and wrist range of motion.

**Table IV:** Size of ulnar styloid fragments in Group 1.

Patient	Ulnar-sided wrist pain
1	2.4
2	3.33
3	3.38
4	3.45
5	3.71
6	4.2
7	4.37
8	4.65
9	4.95
10	4.95
11	5.08
12	5.23
13	5.74
14	6
15	6.46
16	6.91
17	7.09
18	7.31
19	9.21
20	10.02

**Table V:** Sub-group analysis of ulnar styloid fragment size in Group 1.

	Mean fragment size (mm)		p-value
	Absent	Present	
Ulnar-sided wrist pain	4.5	2.4	0.518
ECU tendinitis	4.6	4.1	0.603
DRUJ instability	4.4	4.5	0.952
DRUJ pain	4.3	4.7	0.829
**TFCC grind test**	**3.9**	**7.3**	**0.033**

## Discussion

The clinical significance of a concomitant ulnar styloid fracture in distal radius fractures has not been clearly determined in current literature and there exists conflicting recommendations regarding management options. The main finding in our study was that the presence of an ulnar styloid fracture did not have any impact on ulnar-sided symptoms in patients who underwent surgical fixation of distal radius fractures. However, we found that conservatively managed ulnar styloid fractures with a significant fragment size is likely to result in a positive TFCC grind test result after distal radius fixation.

In our study cohort, the presence of a concomitant ulnar styloid fracture did not result in a difference in outcome after surgical fixation of distal radius fractures, with both groups have similar ulnar sided symptoms, wrist range of motion and DASH scores up to two years post-operatively. DASH score was the only parameter which had a poorer mean result in Group 0, but this did not achieve statistical significance and a larger study may be required to examine this discrepancy. ECU tendinitis was the only parameter which had a trend towards statistical significance, and this could be related to the fibrous union of ulnar styloid fracture fragments as most did not achieve bony union on follow-up radiographs. Okoli et al documented similar results in terms of Quick Disabilities of the Arm, Shoulder, and Hand (QDASH) and Patient-Rated Wrist Evaluation (PRWE) scores but did not include radiological assessment and ulnar sided symptoms in their study^14^. Souer et al found that ulnar styloid base fractures had no significant effect on radiographic evaluation and overall outcome, other than an association with poorer grip strength and less flexion post-operatively. The difference in methodology could account for this difference as they categorised patients with ulnar styloid tip fractures in the group with “no fractures”, which could be a potential confounder^15^. On the contrary, a retrospective study by Ayalon et al found that patients with ulnar styloid fractures had more pain and poorer DASH scores in 184 surgically managed distal radius fractures^16^. However, this did not meet the minimal clinically important difference (MCID) of 10 for DASH score^17^. In a smaller study by Amorosa et al found that 41 out of 58 patients with concomitant ulnar styloid fractures had worse DASH scores which met the MCID of 10, but the study cohort was heterogeneous in nature with mixed treatment types rendered for distal radius fractures^18^. Further, even though selected studies suggest poorer outcomes with concomitant ulnar styloid fractures, there are no studies to date which has shown that fixation of ulnar styloid fractures has resulted in improved outcomes. To our knowledge, this is the only study that includes only surgically managed distal radius fracture, finding that there is no association between concomitant ulnar styloid fractures and radiographic parameters, post-operative outcome scores, as well as ulnar-sided symptoms. After performing a sub-group analysis of patients with concomitant ulnar styloid fractures, we found that a higher incidence of positive TFCC grind test was seen distal radius fractures with larger ulnar styloid fragments. Specifically, if the size of a concomitant ulnar styloid fragment on pre-operative radiographs is measured to be 7.3mm and above, clinicians should perform a TFCC grind test to rule out TFCC injuries. This is supported by the study from Kim et al, where they reported that tears of TFCC foveal insertion occur in 88-89% of distal radius fractures with concomitant ulnar styloid fractures^19^. They also found that complete tear of the TFCC foveal insertion, independent of location of the ulnar styloid fracture, was associated with DRUJ instability, contrary to our finding where there was no difference in DRUJ instability. Unfortunately, our study did not include magnetic resonance imaging (MRI) scans or arthroscopic assessment to establish the diagnosis of any TFCC injuries. This is the first clinical study examining the effects of the size of an ulnar styloid fracture fragment on ulnar-sided wrist pain in surgically managed distal radius fractures, and further studies can examine this parameter instead of the anatomical location of an ulnar styloid fracture.

The ulnar styloid is well established as a strut for soft tissue attachment over the ulnar aspect of the wrist. The base of the ulnar styloid or fovea forms the attachment of the ulnocarpal ligaments, the triangular fibrocartilage complex (TFCC) and the sheath of the extensor carpi ulnaris (ECU). Non-union of ulnar styloid fracture can result in ulnar-sided wrist pain and extensor carpi ulnaris tendinitis^4^, and when the base of the ulnar styloid is involved, the concern lies in the association with disruption of the triangular fibrocartilage complex, as well as distal radioulnar joint (DRUJ) instability^3,20,21^. Ulnar-sided wrist pain in the group without an ulnar styloid fracture was similar to the group without and this could be explained by the presence of DRUJ instability despite an intact ulnar styloid. Thus, the importance of an ulnar-styloid fracture likely lies in its possible association with important soft tissue structures contributing to wrist stability, which likely account for much of the morbidity associated with ulnar styloid fractures. Merely fixing the bony structure itself does not guarantee success. It is also noteworthy TFCC tears and DRUJ instability can occur in the absence of ulnar-styloid fractures. Similar findings were found in a study by Lee *et al* where patients who underwent distal radius fixation without concomitant ulnar styloid fractures had ulnar sided wrist pain, and also had either positive MRI scan findings or intra-operative stress tests, suggesting DRUJ instability^22^. A careful clinical assessment is critical pre-operatively, with due consideration of either performing pre-operative MRI scans or intra-operative wrist arthroscopy in distal radius fractures with ulnar sided symptoms in absence of an ulnar fracture. However, we acknowledge that this could be challenging to discern in the setting of an acute wrist fracture.

We recognise that our study is limited by its retrospective nature, and application of our results may be limited to external institutions as this study was performed in a single institution. Further, our study did not include any radiological or arthroscopic assessment of the TFCC. However, this study should prompt subsequent randomised control studies with a larger study cohort and controlled confounding factors, to investigate the impact of ulnar styloid fragment size on clinical outcomes in surgically managed distal radius fractures.

## Conclusion

In conclusion, the presence or size of ulnar styloid fractures in surgically managed distal radius fractures do not compromise clinical and functional outcomes. However, the presence of an USF of more than 7mm is associated with a positive TFCC grind test.
